# Assessment of Strategies Used in US Hospitals to Address Social Needs During the COVID-19 Pandemic

**DOI:** 10.1001/jamahealthforum.2022.3764

**Published:** 2022-10-21

**Authors:** Jose F. Figueroa, Ciara Duggan, Cristina Toledo-Cornell, Jie Zheng, E. John Orav, Thomas C. Tsai

**Affiliations:** 1 Department of Health Policy and Management, Harvard T.H. Chan School of Public Health, Boston, Massachusetts; 2 Brigham and Women’s Hospital, Boston, Massachusetts

## Abstract

This survey study uses 2020 American Hospital Association data to assess strategies of US hospitals serving vulnerable populations in addressing social needs during the COVID-19 pandemic.

## Introduction

Hospitals may play a critical role in alleviating health inequities by addressing underlying social determinants of health (SDOH). Public interest in addressing social needs during the COVID-19 pandemic has been accompanied by substantial health system investments in SDOH.^1^ Although studies have assessed some hospitals’ efforts to address social needs,^1,2,3^ little is known about the extent to which hospitals that serve vulnerable populations are addressing SDOH. We used 2020 American Hospital Association (AHA) survey data to assess such strategies among rural hospitals, critical access hospitals (CAHs), and safety-net hospitals (SNHs) in the US.

## Methods

The Harvard School of Public Health Institutional Review Board approved this survey study. The AHA obtained respondent informed consent, with the agreement that hospitals would remain anonymous. The study followed the AAPOR reporting guideline.

We used the 2020 AHA Annual Survey Social Determinants of Health Supplement, which surveyed hospitals’ efforts across 3 domains: screening for SDOH, programs/interventions to address SDOH, and community partnerships to address SDOH. The first domain assessed whether hospitals screened across 9 SDOH types. The second domain reported on whether hospitals had programs and/or interventions to address these SDOH. The third domain assessed whether hospitals worked with 14 types of external community partners to address SDOH, participate in community health needs assessments, and implement SDOH initiatives. We created 3 index scores across each domain (the 9 SDOH types and 14 partner types are listed in eMethods 1 in the Supplement). Our sample was limited to acute care hospitals. Scores were weighted for survey response (eMethods 2 in the Supplement). We analyzed separate multivariable linear regression models, with the 3 index scores as dependent outcomes, 3 SDOH hospital characteristics as main estimators (rural/urban, CAH, and SNH), and other characteristics as covariates (size, region, profit status, and teaching status). Adjusted values are presented with their SEM. Analyses were conducted using SAS 9.4 (SAS Institute).

## Results

In this survey study, 2734 of 4295 hospitals (64%) responded to the SDOH items (Table 1). In adjusted analyses, rural hospitals screened for a similar number of social needs (mean [SEM] 4.85 [0.15] vs 5.17 [0.07]; difference, −0.32 [95% CI, −0.65 to 0.01]) compared with urban hospitals but implemented fewer programs and/or interventions to address SDOH (mean 5.15 [0.15] vs 5.74 [0.07]; difference, −0.59 [95% CI, −0.91 to −0.26]) and reported fewer community partnerships (mean 16.95 [0.56] vs 18.29 [0.26]; difference, −1.34 [95% CI, −2.62 to −0.05]; Table 2). The CAHs screened for a similar number of SDOH (mean [SEM] 4.91 [0.15] vs 5.19 [0.08]; difference, −0.28 [95% CI, −0.63 to 0.07]) as non-CAHs but addressed fewer social needs (mean 5.31 [0.14] vs 5.74 [0.08]; difference, −0.43 [95% CI, −0.77 to −0.08]) and reported fewer community partnerships (mean 16.95 [0.54] vs 18.43 [0.30]; difference, −1.48 [95% CI, −2.83 to −0.13]). There were no significant differences between SNHs and non-SNHs; however, SNHs reported fewer community partnerships (mean [SEM] 16.91 [0.49] vs 18.34 [0.26]; difference, −1.43 [95% CI, −2.56 to −0.29]). Cohen’s *d* effect sizes ranged from very small to moderate across hospital characteristics.

**Table 1.** Characteristics of Hospital Respondents and Nonrespondents^a^

Characteristic	Respondents (n = 2734)	Nonrespondents (n = 1561)	*P* value
Size			
Large	381 (13.9)	81 (5.2)	<.001
Medium	1049 (38.4)	574 (36.8)	
Small	1304 (47.7)	906 (58.0)	
Teaching			
Major teaching	203 (7.4)	22 (1.4)	<.001
Minor teaching	882 (32.3)	403 (25.8)	
Nonteaching	1649 (60.3)	1136 (72.8)	
Ownership			
Nonprofit	1951 (71.4)	783 (50.2)	<.001
Public	531 (19.4)	410 (26.3)	
For profit	252 (9.2)	368 (23.6)	
US region			
Northeast	387 (14.2)	130 (8.3)	<.001
Midwest	932 (34.1)	376 (24.1)	
South	963 (35.2)	640 (41.0)	
West	452 (16.5)	415 (26.6)	
Location			
Urban	2170 (79.4)	1084 (69.4)	<.001
Rural	564 (20.6)	477 (30.6)	
Safety-net hospital^b^	818 (29.9)	255 (16.3)	<.001
Critical access hospital	767 (28.1)	571 (36.6)	<.001

^a^ Data are presented as No. (%) of hospitals.

^b^ Safety-net hospital status was defined as a hospital in the top quartile of Disproportionate Share Hospital payment percentage, which reflects hospitals that serve a large number of low-income Medicaid beneficiaries and uninsured individuals.

**Table 2.** Differences in Hospital-Reported Efforts on Screening, Programs/Interventions, and Community Partnerships to Address Social Needs by Hospital Characteristic

Hospital characteristic	Screening for SDOH^a^		Programs/interventions to address SDOH^b^		Community partnerships to address SDOH^c^	
	Mean No. (SEM)^d^	Difference estimate (95% CI)	Mean No. (SEM)	Difference estimate (95% CI)	Mean No. (SEM)	Difference estimate (95% CI)
Service to vulnerable populations						
Rural	4.85 (0.15)	−0.32 (−0.65 to 0.01)	5.15 (0.15)	−0.59 (−0.91 to −0.26)^e^	16.95 (0.56)	−1.34 (−2.62 to −0.05)^e^
Urban	5.17 (0.07)	[Reference]	5.74 (0.07)	[Reference]	18.29 (0.26)	[Reference]
Safety net						
Yes	5.22 (0.13)	0.13 (−0.16 to 0.43)	5.52 (0.12)	−0.15 (−0.43 to 0.14)	16.91 (0.49)	−1.43 (−2.56 to −0.29)^e^
No	5.09 (0.07)	[Reference]	5.66 (0.07)	[Reference]	18.34 (0.26)	[Reference]
Critical access						
Yes	4.91 (0.15)	−0.28 (−0.63 to 0.07)	5.31 (0.14)	−0.43 (−0.77 to −0.08)^e^	16.95 (0.54)	−1.48 (−2.83 to −0.13)^e^
No	5.19 (0.08)	[Reference]	5.74 (0.08)	[Reference]	18.43 (0.30)	[Reference]
Size						
Large	5.03 (0.21)	−0.04 (−0.55 to 0.46)	5.80 (0.20)	0.32 (−0.17 to 0.82)	19.18 (0.80)	2.01 (0.10 to 3.93)^e^
Medium	5.20 (0.10)	0.13 (−0.18 to 0.44)	5.74 (0.10)	0.26 (−0.04 to 0.57)	18.66 (0.39)	1.49 (0.31 to 2.68)^e^
Small	5.07 (0.11)	[Reference]	5.47 (0.10)	[Reference]	17.16 (0.39)	[Reference]
Teaching						
Major teaching	5.57 (0.29)	0.54 (−0.09 to 1.18)	6.71 (0.29)	1.38 (0.76 to 2.00)^e^	21.28 (1.12)	4.05 (1.63 to 6.46)^e^
Minor teaching	5.17 (0.11)	0.14 (−0.15 to 0.43)	5.88 (0.11)	0.56 (0.27 to 0.84)^e^	18.71 (0.43)	1.47 (0.36 to 2.58)^e^
Nonteaching	5.03 (0.09)	[Reference]	5.33 (0.08)	[Reference]	17.24 (0.32)	[Reference]
Ownership						
Nonprofit	5.13 (0.07)	−0.10 (−0.43 to 0.24)	5.84 (0.07)	1.35 (1.02 to 1.68)^e^	19.95 (0.27)	9.07 (7.78 to 10.36)^e^
Public	5.00 (0.13)	−0.23 (−0.62 to 0.15)	5.22 (0.13)	0.74 (0.36 to 1.12)^e^	14.27 (0.50)	3.38 (1.88 to 4.89)^e^
For profit	5.23 (0.15)	[Reference]	4.49 (0.15)	[Reference]	10.88 (0.60)	[Reference]
US region						
Northeast	5.59 (0.16)	0.38 (−0.03 to 0.79)	6.01 (0.16)	0.38 (−0.02 to 0.78)	18.88 (0.63)	0.09 (−1.49 to 1.68)
Midwest	4.92 (0.11)	−0.29 (−0.61 to 0.04)	5.50 (0.10)	−0.13 (−0.45 to 0.19)	17.55 (0.40)	−1.24 (−2.50 to 0.02)
South	5.07 (0.10)	−0.14 (−0.46 to 0.18)	5.58 (0.10)	−0.05 (−0.37 to 0.26)	17.77 (0.38)	−1.02 (−2.25 to 0.22)
West	5.21 (0.13)	[Reference]	5.63 (0.13)	[Reference]	18.79 (0.50)	[Reference]

Abbreviation: SDOH, social determinants of health.

^a^ The screening domain reflects the adjusted difference in the number of SDOH that hospitals reported screening some or most of their patients across 9 types: (1) housing, (2) food insecurity/hunger, (3) utility needs, (4) interpersonal violence, (5) transportation, (6) employment/income, (7) education, (8) social isolation, and (9) health behaviors.

^b^ The programs/interventions domain reflects the adjusted difference in the number of social determinants that hospitals report targeting across SDOH types.

^c^ The community partnerships domain reflects the number of strategies hospitals use to collaborate across 14 different types of external partners on meeting patient social needs, participating in community health needs assessments, and implementing community-level initiatives to address SDOH, which in total result in 42 potential efforts. External partners included (1) health care providers outside their system, (2) health insurance providers outside their system, (3) local or state public health departments and/or organizations, (4) other local or state government agencies or social service organizations, (5) faith-based organizations, (6) local organizations addressing food insecurity, (7) local organizations addressing housing insecurity, (8) local organizations addressing transportation needs, (9) local organizations providing legal assistance for individuals, (10) other community nonprofit organizations, (11) primary and secondary schools, (12) colleges or universities, (13) local businesses or chambers of commerce, and (14) law enforcement and/or safety forces.

^d^ Adjusted means are presented with SEM values.

^e^ Estimated differences with 95% CIs that did not cross 0, reflecting *P* < .05. *R*^2^ estimates from the linear regression models were 3% for the screening domain, 12% for the SDOH interventions/programs domain, and 17% for the community partnership domain.

## Discussion

The results of this survey study suggest that rural hospitals, CAHs, and SNHs are not doing more and, in some cases, are engaging in fewer strategies to address the SDOH of their vulnerable populations, especially regarding community partnerships. This finding may be attributable to limited financial resources, workforce constraints, limited community resources and institutional partnerships, and lack of incentives.^3,4,5,6^

Study limitations include limited granularity of categorial responses of hospital efforts, potential variability and unreliability of respondent accuracy, and limited generalizability. The AHA survey also does not validate whether hospitals implemented the reported strategies or their effectiveness. Addressing resource barriers that hospitals serving vulnerable populations may face in implementing SDOH initiatives should be considered a key state and federal policy strategy for improving health equity.

## Notes

Supplement. **eMethods 1.** Calculating Social Determinants of Health Index Scores From the 2020 American Hospital Association Annual Survey Social Determinants of Health Supplement **eMethods 2.** Construction of Survey Weights for Survey Nonresponse
