# Supplementary material for: Assessment of Strategies Used in US Hospitals to Address Social Needs During the COVID-19 Pandemic
Source: JAMA Health Forum. 2022 Oct 21;3(10):e223764. doi: 10.1001/jamahealthforum.2022.3764 (PMC9587473; doi:10.1001/jamahealthforum.2022.3764)
Supplement: Supplement. — eMethods 1. Calculating Social Determinants of Health Index Scores From the 2020 American Hospital Association Annual Survey Social Determinants of Health Supplement eMethods 2. Construction of Survey Weights for Survey Nonresponse [file jamahealthforum-e223764-s001.pdf]

Supplementary Online Content

Figueroa JF, Duggan C, Toledo-Cornell C, Zheng J, Orav EJ, Tsai TC. Assessment of strategies used in US hospitals to address social needs during the COVID-19 pandemic. *JAMA Health Forum*. 2022;3(10):e223764. doi:10.1001/jamahealthforum.2022.3764

eMethods 1. Calculating Social Determinants of Health Index Scores From the 2020 American Hospital Association Annual Survey Social Determinants of Health Supplement
eMethods 2. Construction of Survey Weights for Survey Nonresponse

This supplementary material has been provided by the authors to give readers additional information about their work.

© 2022 Figueroa JF et al. *JAMA Health Forum*.

eMethods 1. Calculating Social Determinants of Health Index Scores From the 2020 American Hospital Association Annual Survey Supplement

Below, we include the questions as asked in the AHA SDOH Supplement across the 3 SDOH domains. Of note, we have left the exact numbering as it appears in the AHA supplement.

1. SDOH Programs/Interventions Index Score

Section F of the 2020 AHA Annual Survey also included the following item regarding hospitals'/health systems' efforts to address patients' social needs or community SDOH through targeted programs or strategies:

F.1. *Which social needs of patients/social determinants of health in communities does your hospital or health system have programs or strategies to address? (Check all that apply)*

☐ *A. Housing (instability, quality, financing)*
☐ *B. Food insecurity or hunger*
☐ *C. Utility needs*
☐ *D. Interpersonal violence*
☐ *E. Transportation*
☐ *F. Employment and income*
☐ *G. Education*
☐ *H. Social isolation (lack of family and social support)*
☐ *I. Health behaviors*

Calculating Index Score: We calculated an index score for SDOH interventions by summing the total number of social needs addressed by the hospital or health system through targeted programs or strategies as reported in item F.1. (out of 9 possible types of social needs). Possible SDOH intervention index score values therefore ranged from 0 to 9 points.

2. SDOH Screening Index Score

In the 2020 AHA Annual Survey, Section F ("Addressing Patient Social Needs and Community Social Determinants of Health") included the following questions about hospitals/health systems efforts to screen patients for social needs:

F.2. Does your hospital or health system screen patients for social needs?

F.2.a. *If yes, please indicate which social needs are assessed. (Check all that apply)*

☐ *A. Housing (instability, quality, financing)*
☐ *B. Food insecurity or hunger*
☐ *C. Utility needs*
☐ *D. Interpersonal violence*
☐ *E. Transportation*

© 2022 Figueroa JF et al. *JAMA Health Forum*.

- ☐ *F. Employment and income*
- ☐ *G. Education*
- ☐ *H. Social isolation (lack of family and social support)*
- ☐ *I. Health behaviors*

Calculating Index Score: We calculated an index score for SDOH screening by summing the total number of social needs for which the hospital or health system screens its patients, as reported in item F.2.a. (out of 9 possible types of social needs). For hospitals/health systems that responded "Yes, for all patients" to item F.2., each social need reported in F.2.a. was assigned a weight of 1; for those who responded "Yes, for some patients" to item F.2., each social need reported in F.2.a. was assigned a weight of 0.5. Respondents who responded "No (skip to question 3)" on item F.2. were assigned an index score of 0. Possible SDOH screening index score values therefore ranged from 0 to 9 points.

3. SDOH Community Partnerships Index Score

Finally, Section F of the 2020 AHA Annual Survey included the following items to assess the extent to which hospitals/health systems are working with external partners to implement population or community health initiatives:

F.5. *Please indicate the extent of your hospital's current partnerships with external partners for population and/or community health initiatives. Which types of organizations do you currently partner with in each of the following activities? (Check all that apply)*

	Not Involved	***Work together to meet patient social needs (e.g., referral arrangement or case management)***	***Participates in our Community Health Needs Assessment process***	***Work together to implement community-level initiatives to address social determinants of health***
a. *Health care providers outside your system*	☐	☐	☐	☐
b. *Health insurance providers outside of your system*	☐	☐	☐	☐
c. *Local or state public health departments/ organizations*	☐	☐	☐	☐
d. *Other local or state government agencies*	☐	☐	☐	☐

© 2022 Figueroa JF et al. *JAMA Health Forum*.

or social service organizations				
e. *Faith-based organizations*	☐	☐	☐	☐
f. *Local organizations addressing food insecurity*	☐	☐	☐	☐
g. *Local organizations addressing housing insecurity*	☐	☐	☐	☐
h. *Local organizations addressing transportation needs*	☐	☐	☐	☐
i. *Local organizations providing legal assistance for individuals*	☐	☐	☐	☐
j. *Other community non-profit organizations*	☐	☐	☐	☐
k. *K-12 schools*	☐	☐	☐	☐
l. *Colleges or universities*	☐	☐	☐	☐
m. *Local businesses or chambers of commerce*	☐	☐	☐	☐
n. *Law enforcement/safety forces*	☐	☐	☐	☐

Calculating Index Score: We used these items to calculate an SDOH community partnerships index score for each hospital/health system. For each type of external partner (e.g., “a. Health care providers outside your system”), the hospital received 0 to 3 points based on their level of involvement with the partner. Respondents that checked the first option, “Not Involved,” were assigned 0 points. The respondent received 1 point if they indicated that they “work[ed] together to meet patient social needs (e.g., referral arrangement or case management),” an additional 1

© 2022 Figueroa JF et al. *JAMA Health Forum*.

point if they indicated that the partner “participates in [the hospital or health system’s] Community Health Needs Assessment process,” and 1 additional point if they “work together to implement community-level initiatives to address social determinants of health,” for a potential total of 3 points. We repeated this process for each of the 14 types of external partners and summed these points to calculate an SDOH community partnerships index score for each survey respondent. Possible SDOH intervention index score values therefore ranged from 0 to 42 points.

© 2022 Figueroa JF et al. *JAMA Health Forum*.

eMethods 2. Construction of Survey Weights for Survey Nonresponse

To adjust for survey nonresponse rates across hospitals, we first constructed a logistic regression model, in which, responding to the survey was the primary outcome, and hospital characteristics—including hospital size, teaching status, ownership, CAH vs. non-CAH, SNH vs. non-SNH, U.S. region, and urban vs. rural location—were predictors. Each hospital received a likelihood of response based on this model; responses were then weighted with the inverse of this likelihood. In this way, our weighted sample generalizes to all acute general hospitals in the United States.

© 2022 Figueroa JF et al. *JAMA Health Forum*.
